# The Link of the Prion Protein with Ca^2+^ Metabolism and ROS Production, and the Possible Implication in Aβ Toxicity

**DOI:** 10.3390/ijms20184640

**Published:** 2019-09-19

**Authors:** Agnese De Mario, Caterina Peggion, Maria Lina Massimino, Rosa Pia Norante, Alessandra Zulian, Alessandro Bertoli, Maria Catia Sorgato

**Affiliations:** 1 Department of Biomedical Science, University of Padova, 35131 Padova, Italy; agnese.demario@unipd.it (A.D.M.); caterina.peggion@unipd.it (C.P.); rosa.norante@gmail.com (R.P.N.); zulian.alessandra@gmail.com (A.Z.); alessandro.bertoli@unipd.it (A.B.); 2 CNR Neuroscience Institute, Department of Biomedical Science, University of Padova, 35131 Padova, Italy; marilina.massimino@gmail.com; 3 Padova Neuroscience Center, University of Padova, 35131 Padova, Italy

**Keywords:** prion protein, Ca^2+^ homeostasis, glutamate, neurotransmission, synapses, ROS, Aβ oligomers, Fyn, NADPH oxidase

## Abstract

The cellular prion protein (PrP^C^) is an ubiquitous cell surface protein mostly expressed in neurons, where it localizes to both pre- and post-synaptic membranes. PrP^C^ aberrant conformers are the major components of mammalian prions, the infectious agents responsible for incurable neurodegenerative disorders. PrP^C^ was also proposed to bind aggregated misfolded proteins/peptides, and to mediate their neurotoxic signal. In spite of long-lasting research, a general consensus on the precise pathophysiologic mechanisms of PrP^C^ has not yet been reached. Here we review our recent data, obtained by comparing primary neurons from PrP-expressing and PrP-knockout mice, indicating a central role of PrP^C^ in synaptic transmission and Ca^2+^ homeostasis. Indeed, by controlling gene expression and signaling cascades, PrP^C^ is able to optimize glutamate secretion and regulate Ca^2+^ entry via store-operated channels and ionotropic glutamate receptors, thereby protecting neurons from threatening Ca^2+^ overloads and excitotoxicity. We will also illustrate and discuss past and unpublished results demonstrating that Aβ oligomers perturb Ca^2+^ homeostasis and cause abnormal mitochondrial accumulation of reactive oxygen species by possibly affecting the PrP-dependent downregulation of Fyn kinase activity.

## 1. Introduction

Nowadays, neurodegenerative disorders affecting elderly people is one major health problem worldwide, which is aggravated by lack of early diagnostic tools and pharmaceutical approaches. To name a few, they include amyotrophic lateral sclerosis and most common diseases such as Alzheimer’s (AD) and Parkinson′s. Despite its much less occurrence (1–2 cases per million), prion disorders have become renown after appearance of ′mad cow disease′ [1], and the development in man of a variant form of the Creutzfeldt–Jakob disease [2] caused by ingestion of bovine prion-tainted food. All these disorders may have a genetic origin, which, however, accounts for only a few percentages of cases. Typically, they share both a prevalent sporadic etiology and formation of toxic soluble oligomers of misfolded proteins or peptides, which may eventually deposit as amyloids [3]. Remarkably, the physiologic role of most disease-associated proteins is still largely obscure, thus precluding the design of appropriate (mechanism-based) interventions interfering with the neurodegenerative process.

Prion disorders are associated with formation and aggregation in the brain of a template-induced abnormal folding (termed PrP^Sc^) of the constitutively expressed, α helix-rich prion protein (PrP^C^). Mostly present in the pre- and post-synaptic neuronal membranes [4,5,6,7], PrP^C^ localizes to the cell surface through a glycosylphosphatidylinositol (GPI) anchor, and segregates in cholesterol-sphingolipid rich microdomains [8,9]. The β sheet-enriched PrP^Sc^ conformer is the major component of prions causing inevitably fatal diseases in humans and animals [10]. To date, prions are apparently the only neurodegenerative agent retaining the feature of transmissibility within or between mammalian species [3,11]. As to the modality leading to neuronal death, although PrP^Sc^ oligomers could be harmful per se, a growing body of evidence accumulated in in vitro and in vivo paradigms now conceives that the GPI-based tethering of PrP^C^ to the plasma membrane (PM) is strictly required for PrP^Sc^ to unambiguously exert neurotoxicity [12,13,14,15,16]. It is hypothesized that PrP^C^ forms receptor platforms with other surface and/or transmembrane partners engaged in a panoply of downhill beneficial functions, ranging from embryogenesis to cell survival and neuroprotective signaling [17,18,19,20,21]. It is therefore possible to assume that the demonstrated docking of pathogenic prion oligomers to PrP^C^ [22,23] distorts PrP^C^ native structure-function relationship, thus ultimately provoking subversion of advantageous signals into pathways noxious to the cell life.

The present review will first illustrate recent studies by our laboratory aimed to shed light on PrP^C^ physiology, particularly on the action(s) of PrP^C^ in synapses that precisely link the protein with post-synaptic Ca^2+^ fluxes, and downstream events. In addition, the second part of the presentation will provide unpublished in vitro data suggesting that the detrimental effect of AD-related Aβ_1-42_ peptides in neurons may include loss of PrP^C^ protection over Ca^2+^ metabolism and production of reactive oxygen species (ROS).

## 2. PrP^C^ Supports Correct Neuronal Activities by Regulating Gene Expression and Ca^2+^ Metabolism

### 2.1. Action of PrP^C^ at Pre-Synapses

In a recent work [24], we exploited multiple experimental approaches to clarify the role of PrP^C^ at pre-synaptic endings. Specifically, mass spectrometry was used to quantify glutamate release from depolarized primary cerebellar granule neurons (CGN) expressing, or not (PrP-KO), the protein; immunolabeling approaches to follow synaptic vesicle recycling; quantitative mass spectrometry, flanked by selected reaction monitoring and qRT-PCR for data validation, to determining the protein expression profile in the two neuronal types. These analyses clearly demonstrated that glutamate release and synaptic vesicle dynamics are profoundly compromised in PrP-KO CGN (by 40% and 60%, respectively), a result that could be attributed to the underrepresentation of proteins that, among others, are key actors in the two processes—i.e., Rab 3a, syntaxin 1b, and synaptogyrin 3—that are involved in neurotransmitter release, and other three Rab isoforms playing a role in vesicular trafficking. On the other hand, bioinformatic and biochemical approaches demonstrated that PrP^C^ ensured proper gene expression of these proteins by acting on (at least) the transcription factor cAMP-response element binding protein 1 (CREB1) [25], which regulates most of the above genes.

By underscoring the contribution for optimal neurotransmitter secretion, this novel finding undoubtedly defines more accurately the significance of neuroprotective activities long attributed to PrP^C^ [19,20]. At the same time, were such functions disrupted, it may also define that one key molecular pathway possibly contributing to disease phenotypes arises at pre-synapses. As aforementioned, one event likely changing the structure–function coupling of PrP^C^ in prion diseases is the binding of accumulated prions to PrP^C^ [22,26]. As a consequence, prions may displace PrP^C^ from its native PM location, or may substitute its physiologic partners and/or modify their mode of interaction [26]. All these events could thus provoke loss of PrP^C^ ability to properly regulate gene expression and neurotransmission therein. 

Likewise, disastrous synaptic dysfunction and cognitive impairment may be generated by other neurotoxic species equally requiring PrP^C^ as binding receptor; for example, extracellular soluble Aβ oligomers, the 50% of which bind PrP^C^ [27,28,29] (but see [30,31]), and which are AD major initiators [32,33] together with intracellular aggregates of the hyper-phosphorylated tau protein [34]; or α-synuclein oligomers associated with synucleinopathies [35,36].

Intuitively, a critical extension for PrP^C^ capacity to optimize secretion of glutamate, the prime excitatory neurotransmitter in the central nervous system, is that PrP^C^ allows post-synaptic routes for Ca^2+^ entry to be adequately reached. A compromised PrP^C^ function would instead preclude full activation of ionotropic (iGluR) and metabotropic (mGluR) glutamatergic receptors, ultimately resulting in an imbalanced intracellular Ca^2+^ homeostasis and, therefore, in defective Ca^2+^-based processes of learning and memory.

### 2.2. Action of PrP^C^ on SOCE and iGluRs

To decipher other aspects of PrP^C^ operational modes at synapses, we considered past reports on the impact of the protein on Ca^2+^-dependent pathways, and their deviation in prion and PrP-KO paradigms (reviewed in [37,38,39]), but also the ambivalent nature shared by PrP^C^ (see above) and Ca^2+^. Ca^2+^ is a highly versatile transducer of extracellular signals mediating a myriad of cellular responses. Yet it triggers cell demise upon disruption of its homeostasis [40], as observed in neurodegenerative disorders such as AD [41,42]. Specifically, our studies focused on identifying the sites at which the PM-bound PrP^C^ could regulate the entry of extracellular Ca^2+^, and the downstream distribution of the ion, which implied a strategy monitoring Ca^2+^ variations locally. To this end, we prevalently compared the signal of gene-encoded Ca^2+^-sensitive proteins (aequorins) engineered to target specific cell compartments [43] of primary cultures of CGN and cortical neurons expressing, or not, PrP^C^. We used aequorins addressing PM inner microdomains (thus ideally serving to sense the immediate Ca^2+^ transit through the PM [40]), or the bulk cytosol, or the mitochondrial matrix. Such an approach successfully demonstrated that, compared to PrP-KO neurons, the presence of PrP^C^ profoundly attenuates external Ca^2+^ influx through different PM routes, i.e., the process known as store-operated Ca^2+^ entry (SOCE) [44,45], and iGluR-channels [46], as well as the subsequent Ca^2+^ transients in various cell compartments. 

**Figure 1:**
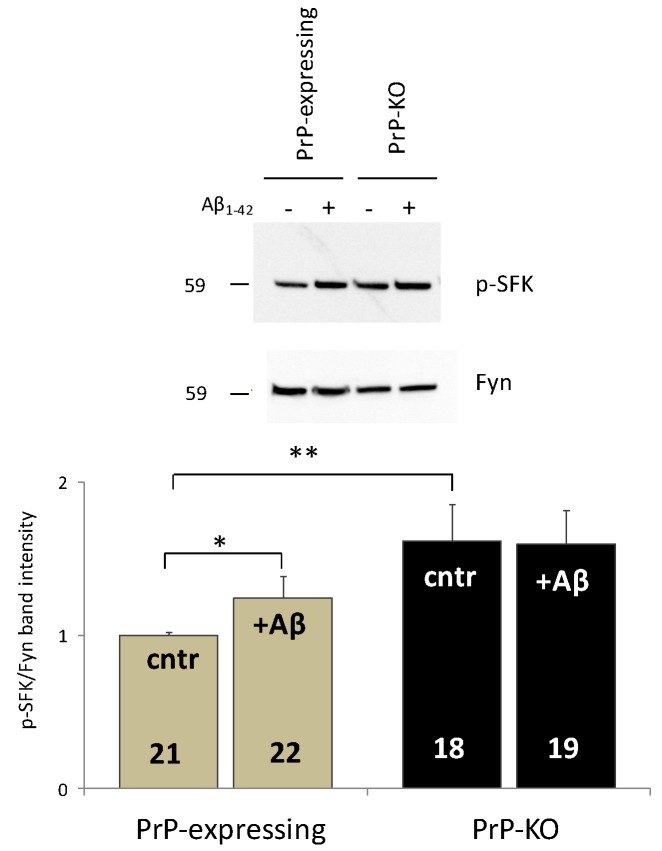
Soluble Aβ_1–42_ oligomers abrogate the PrP-dependent control over Fyn in PrP-expressing primary cortical neurons. Both the representative western blot (WB) obtained after probing PrP-expressing and PrP-KO cortical neurons (treated (+), or not (−), with Aβ_1–42_ oligomers) with antibody to active (auto-phosphorylated, p) SFK or total Fyn (upper panel), and the densitometric analysis of the pSFK immuno-reactive bands normalized to the corresponding signal of total Fyn (lower panel), show that Aβ addition significantly relieves the restriction on Fyn activation observed in untreated PrP-expressing controls relative to PrP-KO counterparts (cntr, grey and black bars, respectively). Conversely, no impact of Aβ was observed in PrP-KO neurons (see text for the explanation of the higher Fyn activation in untreated PrP-KO neurons). After seven days in culture, primary cortical neurons (isolated from PrP-expressing and PrP-KO mice lines Tg46 and F10, respectively [57] (Authorization n. 13/2012), and cultured as previously described [46]), were incubated (1 h, 37 °C), or not, with Aβ_1–42_ oligomers (5 µM, prepared and oligomerized as in [45]), and then lyzed using an ice-cold buffer containing glycerol (10% (*w*/*v*)), SDS (2% (*w*/*v*)), Tris/HCl (62.5 mM, pH 6.8), urea (1.8 M), Na_3_VO_4_ (5 mM), and cocktails of protease and phosphatase inhibitors (Roche). Total protein quantification, WB and densitometric analyses were accomplished as described elsewhere [45]. Here and after, values are expressed as mean ± SEM, and the number of replicates (i.e., different replicates from at least four primary cortical cultures prepared on different days) is indicated inside each bar diagram. * *p* < 0.05, ** *p* < 0.01 (Student’s *t*-test).

In the case of SOCE, this unusual but evolutionary conserved means for Ca^2+^ transport is triggered by depletion (physiological or, in vitro, by adding Ca^2+^ chelators) of endoplasmic reticulum (ER) Ca^2+^ stores, which is sensed by two ER membrane-spanning proteins (stromal interaction molecules, STIM1 and 2 [47,48] but also [45,49,50,51]). Following their conformational change, STIM1/2 migrate and concentrate in areas juxtaposed to the PM favoring interaction with, and opening of a specific Ca^2+^-permeable channel complex allowing SOCE [47]. STIM1 Tyr-phosphorylation by non-receptor Src family kinases (SFK) is crucial for SOCE full activation [48]. Our analyses proved that this event could well explain the lower Ca^2+^ transients (by around 35%) observed in PrP-expressing CGN compared to PrP-KO neurons [45]. Indeed, PrP^C^ strongly downregulated (by around 50%) [45] the SFK member Fyn [52], possibly implicating group-I mGluRs [7,53,54] or other transmembrane proteins [55] to allow PrP^C^ and Fyn communication from the opposite sides of the PM. In turn, PrP^C^-Fyn coupling decreased the amount of active STIM1 in PrP-expressing controls, and, accordingly, of SOCE [50]. Importantly, PrP^C^-driven limitation of Fyn activity was evident both under basal conditions (with Ca^2+^-filled ER stores) and with ER empty stores (as after addition of Ca^2+^ chelators, see above). Another consequence of SOCE attenuation in PrP-expressing neurons was a reduced Ca^2+^ accumulation in the cytosol, and likewise in the mitochondrial matrix [45]. This is important for the cell survival, because Ca^2+^ overflow in the matrix of mitochondria augments the permeability of the inner membrane, which threatens the organelle intactness and the cell life [56]. To note that enhanced SOCE [45], and a more active Fyn (Figure 1), equally pertained to PrP-KO cortical neurons.

When iGluR-channels of CGN were individually activated by specific agonists [*N*-methyl-d-aspartate (NMDA), α-amino-3-hydroxy-5-methyl-4-isoxazole propionic acid (AMPA), or kainate], or simultaneously stimulated by glutamate addition, a picture identical to SOCE-activated neurons was recorded at sub-PM Ca^2+^ ′hot spots′ and in the cytosol [46]. Indeed, the absence of PrP^C^ augmented Ca^2+^ permeation (by a mean of 80%) via iGluRs compared to PrP-expressing counterparts. Similar effects were observed in cortical neurons [46]. As to how PrP^C^ regulates iGluRs, while the ways by which PrP^C^ depresses NMDA-sensitive receptors are known (i.e., interaction with the GluN2D regulatory subunit [58]; modulation of the affinity for the co-agonist glycine [59], we found that PrP^C^ drastically reduces the presence of AMPA-sensitive channels at the cell surface. Such a control of receptor trafficking was likely due to the downregulation of extracellular signal-regulated kinase 1 and 2 (ERK1/2) by PrP^C^ [46]. In fact, being that ERK1/2 is unable to restrict the activity of phosphodiesterases under this condition [60], the resulting low cAMP levels likely reduced the PKA-dependent phosphorylation (at Ser845) of the AMPA-sensitive receptor GluR1 subunit [46], which drives the correct receptor trafficking to the PM only in the active (phosphorylated) form [61].

Intriguingly, however, when testing mitochondrial Ca^2+^ uptake, we observed that only exposure of CGN to glutamate replicated exactly the effect of PrP^C^ presence/absence on iGluRs, whereby a higher Ca^2+^ amount entered mitochondria of PrP-KO neurons [46]. Conversely, the opposite occurred when CGN iGluRs were stimulated individually (by NMDA or AMPA), in which case more Ca^2+^ entered mitochondria of PrP-expressing neurons. Another intriguing observation, collected by electron microscopy analysis of the two CGN populations, was that, on average, mitochondria of PrP-KO CGN were around 40% more detached from the PM (hence from iGluR-mediated Ca^2+^ influx) relative to PrP-expressing neurons. Thus, to understand which Ca^2+^ pool, among those exploited by mitochondria [40], could explain the dependence of mitochondrial Ca^2+^ uptake from the mode of iGluR stimulation, we focused on inositol triphosphate (IP_3_)- and ryanodine-sensitive channels that discharge Ca^2+^ from the ER. These channels deliver Ca^2+^ directly into mitochondria by establishing contacts with the mitochondrial membranes [62]. After excluding that the vanishing amounts of mGluRs detected in primary CGN could have contributed to Ca^2+^ release by activating IP_3_-sensitive channels [46], we concentrated on the signaling module, known as Ca^2+^-induced Ca^2+^-release (CICR) [63,64], which is particularly active in excitable cells and implicates ryanodine-sensitive channels. Such an assumption was proven correct because addition of inhibitory ryanodine concentrations abolished the difference in mitochondrial Ca^2+^ accumulation observed in the two untreated-CGN types. These results led us to conclude that the much higher Ca^2+^ quantity flowing into glutamate-stimulated PrP-KO CGN compared to PrP-expressing counterparts potently activates CICR, and the subsequent mitochondrial Ca^2+^ uptake, in spite of the larger mitochondria-PM distance. Conversely, the much smaller Ca^2+^ amounts entering via NMDA- or AMPA-stimulated iGluRs [46] could have been insufficient to influence CICR in both CGN genotypes, thus favoring an efficient sequestration of Ca^2+^ by mitochondria of PrP-expressing neurons, which are positioned much closer to the PM iGluRs.

Taken together, these observations highlight the central role played by PrP^C^ in controlling major post-synaptic Ca^2+^ entry routes, which PrP^C^ accomplishes by modulating kinase activities (Fyn for SOCE; ERK1/2 for AMPA-sensitive receptor channels). Furthermore, our data emphasize the capacity of PM-bound PrP^C^ to finely tune Ca^2+^ transients in the cytosol and, most importantly, in the mitochondrial matrix by regulating CICR as in the case of CGN. In this way, location of PrP^C^ to post-synaptic density [6,65] strongly contributes to avoid Ca^2+^ overload in the various neuronal compartments and the detrimental consequences therein, e.g., glutamate excitotoxic insults threatening neuronal integrity. Concurrently, PrP^C^ is likely to play a role in finely regulating key functions attributed to SOCE, which include neuronal excitability and gene expression [66,67], such as recruitment of Ca^2+^-dependent transcriptional factors as demonstrated for c-fos and nuclear factor of activated T cells [68]. From all these reviewed data, it is therefore possible to conclude that, from whatever synaptic (pre- or post-) membrane its action originates, PrP^C^ primarily controls post-synaptic Ca^2+^ metabolism and, consequently, Ca^2+^-linked synaptic plasticity. To accomplish the works described above, our experimental approach implied the use of a PrP-KO model to enlighten, by comparison, PrP^C^ physiology, and to mimic (to a certain extent) the consequences of compromised PrP^C^ functions. Yet, it is well known that the chronic ablation of PrP^C^ does not result in evident neuronal phenotypes, which would be expected from a compromised pre-/post-synaptic cross-talk consequent to the reduced glutamate exocytosis. This contradiction could, however, be only apparent, because the higher activity of iGluRs (and SOCE) in these cells likely compensates for the deficient glutamate release, thereby ensuring that an adequate Ca^2+^ signal arrives to post-synaptic PrP-KO neurons.

## 3. Effects of Aβ Oligomers on PrP^C^-Linked Neuronal Controls

While analysing SOCE-mediated Ca^2+^ influx, we tested whether 1 h-treatment with soluble oligomers of Aβ_1-42_ peptides (mostly toxic for the pronounced propensity to aggregate [69]) had any impact on SOCE activity of CGN and cortical neurons. We found that Aβ disrupted the control of PrP^C^ over SOCE but only in PrP-expressing neurons [45], and likewise that over the SOCE-regulator, Fyn kinase ([45], for CGN; Figure 1, for cortical neurons). These results indicate that, irrespective of the neuronal type, a PrP-dependent SOCE-mediated abnormal Ca^2+^ accumulation inside cells could well represent an early dangerous action of Aβ_1-_. Another important conclusion is that, because Fyn and SOCE of PrP-KO neurons are immune from Aβ addition, PrP^C^ needs to be present for Aβ_1–42_ to alter Ca^2+^ fluxes.

**Figure 2:**
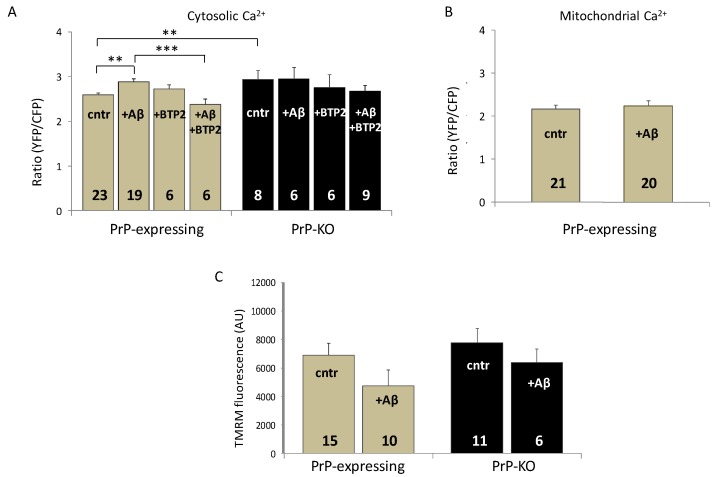
Soluble Aβ_1–42_ oligomers abrogate the PrP-dependent regulation of resting cytosolic Ca^2+^ but does not affect mitochondrial Ca^2+^ levels and membrane potential in PrP-expressing primary cortical neurons. (**A**) Incubation with soluble Aβ_1–42_ oligomers (+ Aβ) increases resting cytosolic Ca^2+^ in PrP-expressing cortical neurons but not in PrP-KO neurons, nullifying the difference observed between the two untreated genotypes (cntr). While preincubation with the SOCE inhibitor *N*-{4-[3,5-bis(Trifluoromethyl)-1*H*-pyrazol-1-yl]phenyl}-4-methyl-1,2,3-thiadiazole-5-carboxamide (+ BTP2, 200 nM) leaves unaffected basal cytosolic Ca^2+^ in untreated cells, and it reverts the Aβ effect in PrP-expressing neurons (+ Aβ, + BTP2), upregulation of SOCE is the likely responsible for the Aβ-induced higher basal Ca^2+^ levels. (**B**) Conversely, Aβ does not affect Ca^2+^ levels in mitochondria of PrP-expressing neurons. Bar diagrams of panels A and B report the ratio between the FRET-acceptor (yellow fluorescent protein, YFP) and -donor (cyan fluorescent protein, CFP) fluorescence intensities of the used cameleon Ca^2+^ probe (D1cpv and 4mtD3cpv [71] in the cytosol and in the mitochondrial matrix, respectively). For these measurements, four days after isolation and plating, neurons were transduced with adeno-associated virus (AAV) coding for the chameleon bearing no targeting sequence (**A**), or a sequence directing the probe to the mitochondrial matrix (**B**), under the control of the pan-neuronal hSyn promoter [75]. Two days after infection, neurons were mounted into an open-topped chamber and maintained in 1 mM CaCl_2_-containing Krebs-Ringer buffer kept at 37 °C using a temperature-controlled apparatus (TC-324B, Warner Instruments). FRET measurements and analysis were performed as previously reported [75]. (**C**) No statistical variation is observed between the mitochondrial membrane potential of untreated, or Aβ-treated, PrP-expressing, and PrP-KO neurons. Here, the bar diagram reports the relative fluorescence quantification of the membrane potential-sensitive probe tetramethylrhodamine methyl ester (TMRM, Molecular Probes) [46] in experiments carried out with neurons loaded with TMRM (20 nM, 30 min, 37 °C) in the presence (+Aβ), or in the absence (cntr), of Aβ_1-42_ oligomers. ** *p* < 0.01, *** *p* < 0.001 (Student′s *t*-test). AU (arbitrary units). Other details are as in the legend to Figure 1.

Next, in light of reports indicating that Aβ strongly impacted on Fyn-mGluR5 coupling, and on several downstream pathways, in PrP-expressing neurons under unstimulated settings [7,70], we replicated these conditions to assess Aβ_1-42_ influence on the resting cytosolic Ca^2+^ concentration (using a particularly sensitive chameleon probe that monitors low basal Ca^2+^ concentrations [71]) of Aβ-treated primary cortical neuronal cultures. As shown (Figure 2A), once again these aggregates were effective only in PrP-expressing neurons by augmenting the Ca^2+^ level up to a quantity similar to that displayed by PrP-KO counterparts. Regarding the molecular basis of this action, we favor the possibility that STIM1—and subsequently SOCE and Ca^2+^ influx—was stimulated by Aβ-induced over-activation of Fyn (Figure 1). Alternatively, or concurrently, one could consider that SOCE activation derived from Aβ ability to deplete ER Ca^2+^ stores, as suggested [72,73]. Whatever the true explanation, SOCE involvement is evident in light of the decreased cytosolic Ca^2+^ following inhibition of the pathway by BTP2 [74] (Figure 2A). 

**Figure 3:**
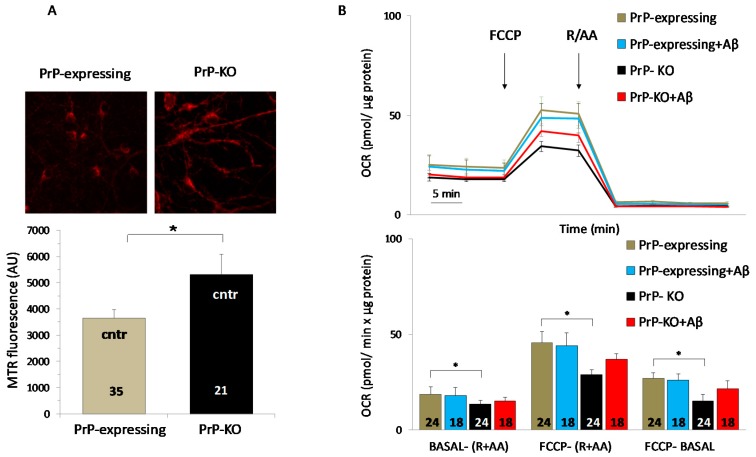
PrP^C^ controls mitochondrial oxygen consumption rate so as to reduce ROS production. (**A**) Higher quantities of reactive oxygen species (ROS) are present in mitochondria of control PrP-KO cortical neurons compared to the PrP-expressing counterparts. This is evident from both the upper panel, showing representative fluorescence micrographs of neurons loaded (30 min, 37 °C) with the ROS-sensitive mitochondrial matrix-accumulating cationic probe MitoTracker Red (MTR, 50 nM (λexc = 579 nm, λem = 599 nm), Molecular Probes), and the lower bar diagram reporting the corresponding MTR fluorescence quantification. Coverslip images were collected using an inverted microscope (Olympus IMT-2) equipped with a Xenon lamp, a 40× oil-immersion objective, appropriate excitation and emission filters and a cooled 16-bit digital CCD camera (Micromax, Princeton Instruments). Following the selection of mitochondria-rich fields in each coverslip, their mean MTR fluorescence was calculated after subtracting the background fluorescence collected in dark fields. (**B**) Compared to PrP-expressing neurons (grey), PrP-KO neurons (black) have a reduced oxygen consumption rate (OCR), as both OCR representative traces (upper panel), and their quantification (lower panel), indicate. Quantification of basal (BASAL-(R + AA)) and maximal (FCCP-(R + AA)) OCR values were calculated by subtracting non-mitochondrial OCR value, obtained after adding mitochondrial respiration blockers antimycin A (AA) and rotenone (R), from the OCR measured, respectively, before and after addition of the respiration uncoupler carbonyl cyanide 4-(trifluoromethoxy)phenylhydrazone (FCCP). Mitochondrial spare respiratory capacity (FCCP-BASAL) was calculated by subtracting the basal OCR value from the maximal OCR value. These results suggest that the slower electron flow by mitochondria of PrP-KO neurons renders more reduced the redox centers of the respiratory chain complexes of these cells, favoring the supply of electrons to oxygen and the production of superoxide anion radicals. However, Aβ-addition does not significantly alter the OCR in either PrP-expressing (light blue) and PrP-KO (red) neurons. For these measurements, neurons were isolated and plated onto XF24 microplate wells (Agilent) for seven days, at the end of which they were treated, or not, with Aβ_1-42_ oligomers. OCR values were real-time assessed with the XF24 Extracellular Flux Analyzer (Agilent), which allows to measure OCR changes after the sequential addition of FCCP (0.5 µM), and antimycin A (1 µM) plus rotenone (1 µM) together. Results were normalized to the protein content determined by a commercial kit (Total Protein Kit, micro Lowry, Peterson′s modification, Sigma). * *p* < 0.05 (Student′s *t*-test). Other details are as in the legend to Figure 1 and Figure 2.

The fact that untreated PrP-KO cortical neurons maintained higher cytosolic Ca^2+^ levels also under basal conditions (Figure 2A) adds further emphasis to the assumption that PrP^C^ controls Ca^2+^ metabolism. An explanation for such a finding may be traced in previous studies showing that PrP-KO CGN express lower amounts of sarcoplasmic/endoplasmic reticulum and PM Ca^2+^ pumps [44], which, therefore, restrict Ca^2+^ extrusion by these neurons relative to when PrP^C^ is present. Plausibly, this is the same reason explaining the higher resting Ca^2+^ observed in PrP-KO cortical neurons. These results prompted us to inspect whether Aβ_1-42_ impacted also on mitochondrial Ca^2+^ fluxes. Figure 2B shows that in this case there was no effect, likely because Aβ-induced Ca^2+^ increase in the cytosol was insufficient to stimulate the mitochondrial uniport complex [62]. A possible additional factor unfavorable to Ca^2+^ uptake is that the membrane potential of mitochondria, and consequently the driving force for Ca^2+^ accumulation, did not significantly vary in both controls (see also [76] and in the presence of Aβ (Figure 2C), as also previously observed [77]. Interestingly, although Aβ oligomers generally depolarize mitochondria of neurons incubated with Aβ for a long period of time [78,79] there are also reports indicating that an acute treatment with Aβ (as was our case) may diminish the membrane potential and/or the oxygen consumption rate [80,81]. Both of these findings are in clear contrast with our observations (Figure 2C and Figure 3B), thereby supporting the possibility that experimental variables (e.g., type of the analyzed model system; type and preparation of the Aβ species, and the added amount, etc.) might be responsible for the contrasting results. We then tested ROS presence in mitochondria using a mitochondrial matrix-accumulating fluorescent probe, following the knowledge that mitochondrial oxidative stress and dysfunctions are common features in several neurological disorders [82,83,84], including AD [85,86,87,88]. One first observation was that, already under control conditions, ROS quantities in mitochondria of PrP-KO cortical neurons were around 45% higher than in PrP-expressing counterparts (Figure 3A). Because mitochondrial respiratory complexes are the major source of free radical production by leaking electrons to molecular oxygen [89], we then inspected mitochondrial oxygen consumption rate (OCR) by the two neuronal populations. The upper and lower panels of Fig. 3B report that PrP-KO cortical neurons exhibited an OCR value around 35% lower than PrP-expressing neurons, both under coupled and uncoupled (after addition of the protonophore FCCP) conditions. These results suggest that the enhanced production of oxygen radicals by mitochondria of control PrP-KO neurons (Figure 3A) may originate from the partially inhibited electron flow that, by rendering more reduced the redox centers of the respiratory complexes, favors their supply of electrons to oxygen and consequently the production of superoxide anion radicals. Albeit conducted under different experimental settings (e.g., polarographic detection of oxygen consumption by isolated brain mitochondria; use of quantitative electron paramagnetic resonance spectroscopy to assess superoxide production by brain submitochondrial particles), a previous study similarly found an increased generation of superoxide radicals in the absence of PrP^C^, which was imputed to a more reduced Complex I relative to the downstream respiratory chain components [90]. Yet, in contrast to our data, a similar respiratory rate under coupled conditions was observed in mitochondria derived from PrP-expressing, and PrP-KO, brains.

**Figure 4:**
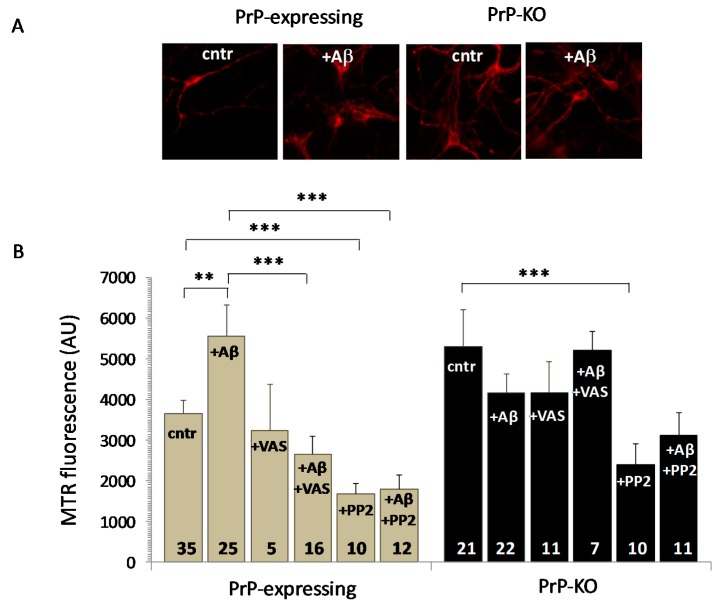
Aβ_1–42_ causes a PrP-dependent mitochondrial ROS overload in PrP-expressing neurons through activation of NADPH oxidase. (**A**,**B**) Starting from the higher ROS quantity present in mitochondria of untreated PrP-KO neurons than in the PrP-expressing counterparts (cntr), Aβ addition (+ Aβ) increases mitochondrial ROS only in PrP-expressing neurons as clearly demonstrated by the representative fluorescence micrographs of neurons loaded with the mitochondrial ROS-sensitive dye MTR and acquired with a 40× objective (**A**), and the bar diagram reporting MTR fluorescence quantification (**B**). The source of such an Aβ-dependent supplementary ROS content in mitochondria of PrP-expressing neurons is the cytosolic NADPH oxidase, in light of ROS diminution upon inhibiting the enzyme with 3-benzyl-7-(2-benzoxazolyl)thio-1,2,3-triazolo(4,5-d)pyrimidine (VAS 2870, 10 µM, 1 h, 37 °C) (+ Aβ + VAS). Also addition of the Fyn inhibitor 3-(4-chlorophenyl) 1-(1,1-dimethylethyl)-1*H*-pyrazolo(3,4-d)pyrimidin-4-amine (PP2, 10 µM, 1 h, 37 °C) reduces ROS presence in mitochondria of PrP-expressing neurons, either in the absence (+PP2) or the presence of Aβ oligomers (+ Aβ + PP2). This is consistent with the uphill stimulation of NADPH oxidase by Fyn and the relieve of the PrP-dependent block of Fyn activation by Aβ oligomers (Figure 1). However, the drastic ROS diminution observed in PrP-KO neurons treated with PP2 (+ PP2), but not with VAS (+ VAS), indicates that other Fyn-controlled (and PrP- and NADPH oxidase-independent) pro-oxidant pathways contribute to mitochondrial ROS accumulation. ** *p* < 0.01, *** *p* < 0.001 (Student′s *t*-test). Other details are as in the legend to Figure 1 and Figure 2.

However, although Aβ_1–42_ oligomers did not significantly alter mitochondrial OCR in both neuronal genotypes (Figure 3B), nonetheless they once again consistently increased mitochondrial ROS only in PrP-expressing cells (Figure 4A,B).

Taken together, these results argue that Aβ-induced over-accumulating ROS in mitochondria likely originated mainly from sources outside the organelle. By NADPH oxidase, for example, the principal cytosolic ROS producer implicated in many neurodegenerative disorders [91], and already identified as a PrP^C^ target [92]. Data of Figure 4B substantiate this possibility because Aβ-induced mitochondrial ROS overload in PrP-expressing neurons was abolished following inhibition of the enzyme by the specific drug VAS 2870 [93]. According to the literature, however, it is also possible that there is a contribution based on the crosstalk between NADPH oxidase-produced ROS and mitochondria (and vice versa) [94], which may stimulate a mitochondrial production of ROS via the ROS induced-ROS release mechanism [95,96]. Even so, taking into account that: (i) the SFK inhibitor PP2 [97] profoundly reduced Aβ-induced ROS presence in mitochondria, irrespective of Aβ addition and of the PrP genotype (Figure 4B); (ii) Aβ over-activates Fyn in PrP-expressing neurons by removing the PrP-dependent constitutive limitation on the kinase (Figure 1); (iii) Fyn is responsible for stimulating NADPH oxidase, possibly through the PKC pathway [92,98], it can be inferred that Aβ_1-42_ signaling requires PrP^C^ for NADPH oxidase-dependent mitochondrial ROS surplus. Finally, considering that Fyn inhibition also decreased mitochondrial ROS of PrP-KO neurons, one can envisage Fyn as a multiple signal-integrating hub [55] amplifying other PrP-independent pro-oxidant pathways that may also include mitochondrial superoxide production [99].

In summary, the capacity of Aβ_1–42_ to bypass the PrP-dependent regulation of Fyn argues that, downhill to PrP^C^ docking, Aβ exploits Fyn to increase cytosolic Ca^2+^ and mitochondrial oxidative stress, either of which may be prodromic to more aggressive insults endangering neuronal survival. 

## 4. Conclusions

Having illustrated our studies dedicated to the physiology of PrP^C^, here we have highlighted that PrP^C^ controls the release of neurotransmitters and post-synaptic Ca^2+^ entry, and protects neurons from ROS. 

In particular, we have discussed the novel finding that PrP^C^ plays an important role in pre-synaptic membranes by optimizing glutamate exocytosis. Glutamate is the main excitatory neurotransmitter responsible for learning and memory [100,101], whose Ca^2+^-mediated signal reaches post-synapses by activating i- and m-GluRs. Regarding iGluRs, our work has not only confirmed with different methodologies previous findings on PrP^C^ capacity to attenuate the activity of NMDA-sensitive iGluRs, but has also demonstrated that the other (AMPA- and kainite-sensitive) iGluR sub-types fall under the same kind of control. Likewise, PrP^C^ limits the activity of SOCE.

These results clearly imply that the operational framework of PrP^C^ spans the entire synaptic junction. Although employing different means (regulation of gene expression at the pre-synaptic membrane; modulation of kinase activities at the post-synaptic membrane), the action of PrP^C^ at the two synaptic sides is fully coherent under the notion of PrP^C^ neuroprotective attributes. On one hand, the protein guarantees that the refined molecular machinery deputed to glutamate secretion (and possibly other neurotransmitters) operates optimally. On the other hand, PrP^C^ also acts as a safeguard against excessive neuronal Ca^2+^ rise at the post-synaptic level (via, e.g., SOCE and iGluRs).

Although other PrP-dependent dysfunctions have been implicated in prion pathology [20,21,102], the here-reviewed data allow to predict that, once PrP^C^ physiologic functions are acutely challenged, the favorable control of the protein over the above pre-/post-synaptic events gets irremediably broken provoking synapse deterioration and loss, which are well established markers of neurodegenerative processes [103]. Excitotoxicity mediated by oxidative stress and increased intracellular Ca^2+^ (for a review, see [104]) are also induced by Aβ oligomers. Having demonstrated that Aβ_1-42_ oligomers augment PM Ca^2+^ fluxes, the resting cytosolic level of the ion, as well as mitochondrial Ca^2+^ and ROS in PrP-expressing neurons, these data favor the contention that Aβ_1-42_ neurotoxic species perturb the normal protective activity of PrP^C^ over Ca^2+^ metabolism and ROS production. Concurrently, they also highlight that PrP^C^ assists Aβ in challenging neuronal integrity.

## Abbreviations

AA	antimycin
AD	Alzheimer′s disease
AMPA	α-amino-3-hydroxy-5-methyl-4-isoxazole propionic acid
BTP2	*N*-{4-[3,5-bis(Trifluoromethyl)-1*H*-pyrazol-1-yl]phenyl}-4-methyl-1,2,3-thiadiazole-5-carboxamide,
CFP	cyan fluorescent protein
CGN	cerebellar granule neurons
CICR	Ca^2+^-induced Ca^2+^-release
CREB	cAMP-response element binding protein
ER	endoplasmic reticulum
ERK	extracellular signal-regulated kinase
FCCP	carbonyl cyanide 4-(trifluoromethoxy)phenylhydrazone
GPI	glycosylphosphatidylinositol
iGluR	ionotropic glutamatergic receptor
IP3	inositol triphosphate
mGluR	metabotropic glutamatergic receptor
MTR	MitoTracker Red
NMDA	N-methyl-D-aspartate
OCR	oxygen consumption rate
PM	plasma membrane
PP2	3-(4-chlorophenyl) 1-(1,1-dimethylethyl)-1*H*-pyrazolo(3,4-d)pyrimidin-4-amine
PrPC	cellular prion protein
PrP-KO	PrP-knockout
PrP^Sc^	abnormal PrP^C^ conformer present in prions
R	Rotenone
ROS	reactive oxygen species
SEM	standard error of the mean
SFK	Src family kinases
SOCE	store-operated Ca^2+^ entry
STIM	stromal interaction molecules
TMRM	tetramethylrhodamine methyl ester
VAS 2870	3-benzyl-7-(2-benzoxazolyl)thio-1,2,3-triazolo(4,5-d)pyrimidine
YFP	yellow fluorescent protein
WB	western blot
